# Human Tau Expression Does Not Induce Mouse Retina Neurodegeneration, Suggesting Differential Toxicity of Tau in Brain vs. Retinal Neurons

**DOI:** 10.3389/fnmol.2018.00293

**Published:** 2018-08-24

**Authors:** Léa Rodriguez, Julius Baya Mdzomba, Sandrine Joly, Mélissa Boudreau-Laprise, Emmanuel Planel, Vincent Pernet

**Affiliations:** ^1^CUO-Recherche, Centre de Recherche du CHU de Québec, Quebec, QC, Canada; ^2^Département d’ophtalmologie, Faculté de Médecine, Université Laval, Quebec, QC, Canada; ^3^Axe Neurosciences, Centre de Recherche du CHU de Québec, Quebec, QC, Canada; ^4^Département de Psychiatrie et de Neurosciences, Faculté de Médecine, Université Laval, Quebec, QC, Canada

**Keywords:** human Tau, Alzheimer’s disease, retina, electroretinogram, photoreceptors, bipolar cells, mTOR

## Abstract

The implication of the microtubule-associated protein (MAP) Tau in the ocular manifestations of Alzheimer’s disease (AD) is elusive due to the lack of relevant animal model. However, signs of AD have been reported in the brain of transgenic mice expressing human Tau (hTau). To assess whether hTau is sufficient to induce AD pathogenesis in the retina as well, in the present study, we compared the retinal structure and function of KO mice deprived of Tau (mTKO) with those of transgenic mice expressing hTau. Our results revealed that hTau is particularly abundant in the inner nuclear layer (INL) cells of the retina. By electroretinogram (ERG) recording, light-induced retinal cell activation was not altered in hTau compared with mTKO littermates. Surprisingly, the ERG response mediated by cone photoreceptor stimulation was even stronger in hTau than in mTKO retinae. Immunofluorescent analysis of retinal sections allowed us to observe thicker inner retina in hTau than in mTKO eyes. By Western Blotting (WB), the upregulation of mTOR that was found in hTau mice may underlie retinal structure and function increases. Taken together, our results not only indicate that hTau expression is not toxic for retinal cells but they also suggest that it may play a positive role in visual physiology. The use of hTau may be envisaged to improve visual recovery in ocular diseases affecting the retinal function such as glaucoma or diabetic retinopathy.

## Introduction

Alzheimer’s disease (AD) is the most common form of dementia characterized by severe cognitive impairments (Perrin et al., 2009). However, visual impairments have also been described in AD patients as reflected by the reduction of visual acuity, contrast sensitivity, color vision, visual field and pupillary function (Uhlmann et al., 1991; Hutton et al., 1993; Gómez-Tortosa et al., 1996; Whittaker et al., 2002). Visual deficits may involve neuronal cell degeneration in the brain (Yan et al., 2017) and in the retina. Indeed, amyloid-β (Aβ) deposits and neurofibrillary tangles (NFTs) containing hyperphosphorylated Tau are classical histopathological features of AD that could also be observed in the retina of patients (Hart et al., 2016; Koronyo et al., 2017; Kusne et al., 2017; van Wijngaarden et al., 2017). The degeneration of retinal ganglion cells (RGCs), the output retinal neurons, has been linked to the upregulation of Aβ and phospho-Tau (Blanks et al., 1991; Parisi, 2003), two molecules that may be responsible for conscious and non-conscious vision impairments in AD. Other retinal cells expressing amyloid precursor protein (APP) and Tau, such as photoreceptors and interneurons, may be affected by AD and thus contribute to visual loss (Elachouri et al., 2015; Chiasseu et al., 2016; Aboelnour et al., 2017). Although the implication of Aβ in retinal dysfunction is well established (La Morgia et al., 2011, 2017; Koronyo-Hamaoui et al., 2011), very little is known on the role that Tau may play in the development of visual deficits linked to AD pathogenesis.

In order to investigate the neurodegenerative processes of AD, different transgenic mouse models expressing human Aβ and Tau have been generated (Ho et al., 2012; Parnell et al., 2012). Mouse lines exhibit typical age-dependent deposit of extracellular Aβ and intracellular NFTs in the cerebellum, hippocampus and cortex correlating with the development of cognitive alterations (Hsiao et al., 1996; Baglietto-Vargas et al., 2014; van Eersel et al., 2015). The most striking AD phenotypes have been obtained with mice expressing multiple human genes allowing Aβ aggregates and NFT formation in the brain. For example, combined expression of mutant APP, Tau and Presenilin1 in 3xTg-AD mice produces progressive RGC degeneration, Aβ deposition and Tau phosphorylation in a similar fashion to what can be observed in the brain (Perez et al., 2009; Edwards et al., 2014; Chiasseu et al., 2017). Unraveling the respective contribution of Aβ and Tau to AD in the retina requires the use of single transgenic models however. In this regard, the appearance of Aβ deposits and the establishment of functional and histological changes is controversial in the retina but not in the cortex or the hippocampus of APPswe/PS1ΔE9 transgenic mouse, a classical AD model (Ning et al., 2008; Dutescu et al., 2009; Perez et al., 2009; Koronyo-Hamaoui et al., 2011; Leinonen et al., 2016; Joly et al., 2017). This suggests that retinal and brain neurons may differently synthesize Aβ, at least in this model. On the other hand, P301S Tau gene mutation causes early-onset frontotemporal dementia with parkinsonism linked to chromosome 17 (FTDP.17), an aggressive form of tauopathy (Sperfeld et al., 1999). The expression of P301S Tau in transgenic mice gives rise to Tau inclusion and neuronal degeneration in brain structures, and to RGC dysfunction in the retina (Allen et al., 2002; Gasparini et al., 2011; van Eersel et al., 2015; Mazzaro et al., 2016). However, P301S MAPT gene mutation is not involved in AD. Alternatively, non-mutant human Tau (hTau) gene expression may be sufficient to initiate AD in mice and may thus constitute a more relevant model to study the disease. Indeed, it has been shown that replacing murine Tau by hTau induced NFTs and cell death in the enthorinal cortex and in the hippocampus of so-called hTau transgenic mice (Andorfer et al., 2003; Andorfer, 2005). These later mice may represent a minimal model for the study of Tau in the mechanisms of AD, without ectopic and exaggerated expression of mutant Tau. Although visual function changes have not been examined in hTau mice during aging, the expression of hTau was reported to cause Tau hyperphosphorylation and severe retina degeneration in the eye of *Drosophila melanogaster* (Wittmann et al., 2001; Steinhilb et al., 2007). These results raise the possibility that the retina of aging hTau mice may undergo similar neurodegeneration and may then be used as a model tissue to better understand the mechanisms of AD.

In the present study, we examined functional, histological and molecular changes occurring in the visual system of hTau mice at 5 and 17 months of age. Electroretinogram (ERG) recordings revealed that the retinal response of hTau mice to light stimulation was not altered compared to that of KO mice deprived of Tau (mTKO). Unexpectedly, our results indicate that the retinal activity mediated by blue cone photoreceptor (S-cones) activation was enhanced in hTau mice relative to mTKO mice. In a consistent manner, our histological observations showed that the inner retina was thicker in hTau than in mTKO mice, presumably due to mTOR signaling activation. Altogether, our data suggest that hTau expression is not detrimental for the function and survival of mouse retinal cells. Instead, hTau may participate in the mouse retinal physiology by modulating mTOR signaling pathway, in respect with mTKO animals.

## Materials and Methods

### Animals

Tau KO mice (mTKO) were generated by inserting the coding sequence of enhanced green fluorescent protein (EGFP) in exon 1 of *Mapt*, hence resulting into *Mapt* gene expression disruption and in the expression of a fused protein composed of EGFP and the first 31 amino acids of MAPT (Tucker et al., 2001). The expression of EGFP driven by mouse Tau promoter could thus be monitored by Western Blotting (WB) in retinal or brain homogenates from mTKO mice. The founders of our mTKO and hTau mice colony (Bar Harbor, ME, USA; B6.Cg-Mapttm1 (EGFP) Klt Tg(MAPT)8cPdav/J) were from the Jackson Laboratory on C57BL/6J background. HTau mice were initially generated in the laboratory of Dr. P. Davies (Andorfer et al., 2003) by crossing mTKO mice with 8c mice that express hTau transgene derived from a human PAC containing the coding sequence, intronic regions and regulatory regions of the human gene (Duff et al., 2000). HTau mice physiologically express the six isoforms of hTau, but do not express mouse tau. These mice develop Tau pathology in a time course and in brain regions comparable to that occurring in the early stages of human AD. Here, 5- and 17-month-old mice of either sex were used (respectively mTKO 5 months, *n* = 4 females and *n* = 2 males—hTau 5 months, *n* = 4 females and *n* = 3 males—mTKO 17 months, *n* = 4 females—hTau 17 months, *n* = 1 female and *n* = 6 males). This study was carried out in accordance with the principles of the Basel Declaration and recommendations of the Canadian Council on Animal Care guidelines. The protocol was approved by the “*comité de protection des animaux du CHUQ-Université Laval*,” the Université Laval Animal Welfare Committee.

### Electroretinography

ERGs were recorded in mice at 5 and 17-months of age with a Ganzfeld ERG system (Phoenix Research Labs, Pleasanton, CA, USA). As previously described by Joly et al. (2017), scotopic and photopic ERGs were recorded in mTKO and hTau littermates. For scotopic recordings, mice were adapted to complete darkness for 12 h beforehand. Under dim red light, mice were anesthetized with an intraperitoneal injection of a mixture of ketamine (10 mg/kg) and xylazine (1 mg/kg). Pupils were dilated with 1% Mydriacyl tropicamide (Alcon) drops. Mice were then installed on a platform covered by a heating blanket to maintain their body temperature during the whole ERG recording session. To prevent corneal dessication and to establish contact between the cornea and the electrode (gold-plate objective lens), a moisturizing solution of 2.5% hypromellose (Goniosoft, OCuSOFT Inc.) was applied on both eyes soon after anesthesia. The reference and the ground electrodes (platinum needles) were subcutaneously inserted on top of the head and into the tail, respectively. Scotopic full-field ERGs (bandwidth: 2–1,000 Hz) were obtained in response to increasing flash intensities, ranging from −4.7 log cd.s.m^−2^ to 2.5 log cd.s.m^−2^ (interstimulus interval, 20 ms; flash duration, 1 ms; 0.6 log-unit increment). Photopic ERGs were recorded just after scotopic recordings. The mouse eye was exposed to constant green light (504 nm; 1.6 log cd.s.m^−2^) for 10 min to saturate rod photoreceptors. Two series of recordings were then performed with green light flashes (504 nm; interstimulus interval, 20 ms; flash duration, 1 ms; average of 20 flashes, from 1.0 log cd.s.m^−2^ to 2.8 log cd.s.m^−2^; 0.6 log-unit increment) and UV light flashes (365 nm; interstimulus interval, 20 ms; flash duration, 1 ms; average of 20 flashes, from 1.0 log cd.s.m^−2^ to 2.8 log cd.s.m^−2^; 0.6 log-unit increment) to selectively excite the M-cone and the S-cone pathways. For scotopic ERG analysis, the amplitude of the a-wave was measured from baseline to the most negative trough, whereas that of the b-wave was measured from the trough of the a-wave to the most positive peak of the retinal response (Joly et al., 2006). Implicit times were calculated from flash onset to peak. Scotopic luminance-response function curves were obtained by plotting the amplitude of the b-wave as a function of the flash intensity used to evoke the response. Statistical analysis was performed by applying nonparametric Mann-Whitney *t*-test (GraphPad Prism, GraphPad Software, La Jolla, CA, USA).

### Immunofluorescence

Mice received a lethal dose of a mixture of ketamine (90 mg/kg) and xylazine (10 mg/kg) and were intracardially perfused with 4% paraformaldehyde (PFA). For retinal sections, after removal of the cornea and the lens, eyecups were cryoprotected in 30% sucrose and embedded in Optimal Cutting Temperature (Cedarlane, Burlington, ON, Canada) medium before cryosectioning (14-μm thick). Tissues sections were collected on Superfrost microscope glass slides. Primary antibodies were diluted in a blocking solution composed of PBS containing 0.3% Triton X-100 and 5% bovine serum albumin (BSA). Retinal cryosections were incubated with primary antibodies overnight at 4°C (see Table 1) and, after washings in PBS, were then incubated with the appropriate secondary antibodies for 1 h at room temperature. Slides were then mounted with the Vectashield mounting medium (Vector Laboratories, Burlington, ON, Canada). Dapi was used as a nuclear marker. Immunofluorescent stainings were analyzed with an LSM 700 laser scanning confocal microscope (Zeiss, Germany), equipped with the ZEN2 software for image acquisition and processing. To determine cell survival and cell densities, mosaic pictures were taken with an Axio Imager M2 microscope connected to an AxioCam Camera using ZEN2 software (Zeiss, Germany). Retinal thickness and cell numbers were counted every 200 μm by using the ImageJ software (Rasband, W.S., ImageJ, US National Institutes of Health, Bethesda, MD, USA, 1997–2016), and data were analyzed with the GraphPad Prism software.

**Table 1:** Antibodies used for immunofluorescence (IF) and western blotting (WB).

Name	Type	Epitope	Dilution IF	Dilution WB	Source	Catalog number
Anti-Tau	rAb	Total Tau 243–441	1:500	1:10,000	Dako	A002401-2
Anti-P.Tau	mAb (PHF1)	pSer396, pSer404		1:1,000	Generous gift from Prof. P. Davies (The Feinstein Institute for Medical Research, Manhasset, NY, USA)	
Anti-P.Tau	mAb (AT8)	pSer202, pThr205	1:200	1:1,000	Thermo Fisher Scientific (Burlington, ON, Canada)	MN1020
Anti-RBPMS	gpAb		1:500		PhosphoSolutions	1832-RBPMS
Anti-PKCα	mAb	373–672	1:200	1:1,000	Santa Cruz (Dallas, TX, USA)	Sc-17769
Anti-PKCα	rAb	373–672	1:200	1:1,000	Santa Cruz	Sc-10800
Anti-SWL	gtAb		1:200	1:2,000	Santa Cruz	Sc-14363
Anti-MWL	rAb		1:500	1:5,000	EMD Millipore	AB5405
Anti-Recoverin	rAb		1:500	1:2,000	EMD Millipore	AB5585
Anti-P.mTOR	rAb	Ser2448	1:50	1:1,000	Cell Signaling (Whitby, ON, Canada)	5536
Anti-mTOR	rAb	Ser2481		1:1,000	Cell Signaling	2983
Anti-P.AKT	rAb	Ser473		1:1,000	Cell Signaling	9271
Anti-AKT	rAb	Ser473		1:1,000	Cell Signaling	9272
Anti-P.S6	rAb	Ser235-Ser236	1:100	1:1,000	Cell Signaling	4858
Anti-P.STAT3	rAb	Tyr705		1:500	Cell Signaling	9131
Anti-STAT3	rAb	Gly700		1:1,000	Cell Signaling	D3Z2G
Anti-β-actin	mAb			1:5,000	Sigma (Oakville, ON, Canada)	A5441

*Abbreviations: Ab, antibody; m, mouse; r, rabbit; gt, goat; gp, guinea pig*.

### Western Blot Analysis

Retinae were quickly dissected, snap frozen in liquid nitrogen and stored at −80°C until protein extraction. Retinae were homogenized for 1 h on ice in Eppendorf tubes containing fresh lysis buffer (20 mM Tris-HCl, 0.5% CHAPS, pH 8.0) and protease and phosphatase inhibitor pellets (Thermo Scientific, Wilmington, DE, USA). Lysate samples were centrifuged for 15 min at 15,000× *g* at 4°C. Supernatants were then collected and used to determine protein concentration (BioRad, Mississauga, ON, Canada). Proteins (20 μg/lane) were resolved by electrophoresis on a 4%–12% gradient polyacrylamide gel and transferred to nitrocellulose membranes. Nitrocellulose membranes were pre-incubated in a blocking solution of 5% BSA dissolved in TBST (Tris-base 0.1 M, 0.2% Tween 20, pH 7.4) for 1 h at room temperature, incubated with primary antibodies under agitation overnight at 4°C. After washes, membranes were re-incubated with a horseradish peroxidase-conjugated anti-mouse or anti-rabbit antibody (1:10,000; Pierce Biotechnology, Burlington, ON, Canada). Primary antibodies are presented in Table 1. Chemiluminescent bands were detected with LiCor Western Sure Premium Chemiluminescent Substrate (Mandel, Guelph, ON, Canada) in a LiCor C-Digit blot scanner (Mandel). Band signals were quantified with the ImageJ software and analyzed with the GraphPad Prism software.

### Histological Analysis of Hippocampal Brain Sections at 17-Months of Age

After fixation with 4% PFA and cryoprotection in 30% sucrose, four hippocampal sections from 17-month-old mTKO and hTau brains (*n* = 3 per group) were cut (20-μm thick) between Bregma −1.55 mm and −2.27 mm and stained with Dapi overnight at 4°C. Slices were then mounted with Vectashield mounting medium (Vector Laboratories, Burlington, ON, Canada). Mosaic pictures were captured with an Axio Imager M2 microscope connected to an AxioCam Camera using ZEN2 software (Zeiss, Germany). The thickness of the CA1 pyramidal cell layer (CA1) and of the dentate gyrus (DG) granular cell layer were measured at two (CA1) and three (DG) spots on each slice with the ImageJ software (NIH). Average values were calculated in three animals per genotype.

### Statistical Analysis

Data are presented as mean ± standard error of the mean (SEM). Statistical analyses were performed with the GraphPad Software, Prism 5; GraphPad Software Inc., San Diego, CA, USA by applying an unpaired, non-parametric *t*-test to assess statistical significance when only two experimental groups were compared such as for Western Blot analysis and immunofluorescence (IF) intensity measurements (Figures 2–5, Supplementary Figure S3). In experiments comprising more than two groups, analysis were performed by applying one-way analysis of variance (ANOVA) tests followed by the Tukey’s *post hoc* test (Figure 6).

**Figure 1:**
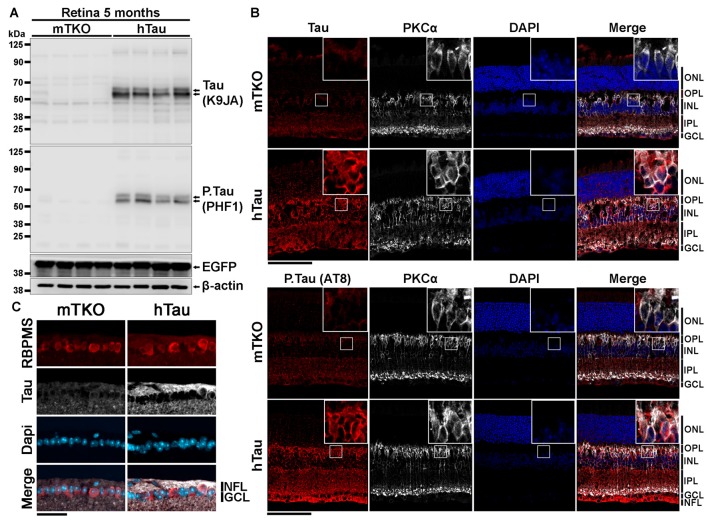
Characterization of human Tau (hTau) expression in transgenic mouse retinae. **(A)** By western blotting (WB), total Tau (K9JA), phosphorylated Tau (P.Tau; PHF1) and enhanced green fluorescent protein (EGFP) protein expression was analyzed in retinal lysates of 5-month-old KO mice deprived of Tau (mTKO) and hTau mice. Tau and P.Tau were expressed in hTau mice but not in mTKO mice. Tau promoter-driven EGFP expression was similar in the two mouse genotypes. **(B)** The distribution of Tau expression in retina was performed by Immunofluorescence (IF) on retinal sections using K9JA (total Tau), AT8 (P.Tau). The localization of Tau was determined by co-staining retinal cryosections for Tau or P.Tau and PKCα, a bipolar cell marker. Retinal layer nuclei were labeled with DAPI. The hTau retinae presented a strong staining for Tau or P.Tau in the NFL, GCL, IPL, INL and ONL contrary to mTKO retinae where no staining was observed. **(C)** Retinal ganglion cells (RGCs) were visualized in the GCL using RBPMS as a marker. Tau was predominantly located in the axonal compartment of those neuronal cells, in the NFL. Scale bars: **(B)** = 100 μm, **(C)** = 50 μm. Abbreviations: NFL, nerve fiber layer; GCL, ganglion cell layer; INL, inner nuclear layer; IPL, inner plexiform layer; ONL, outer nuclear layer.

**Figure 2:**
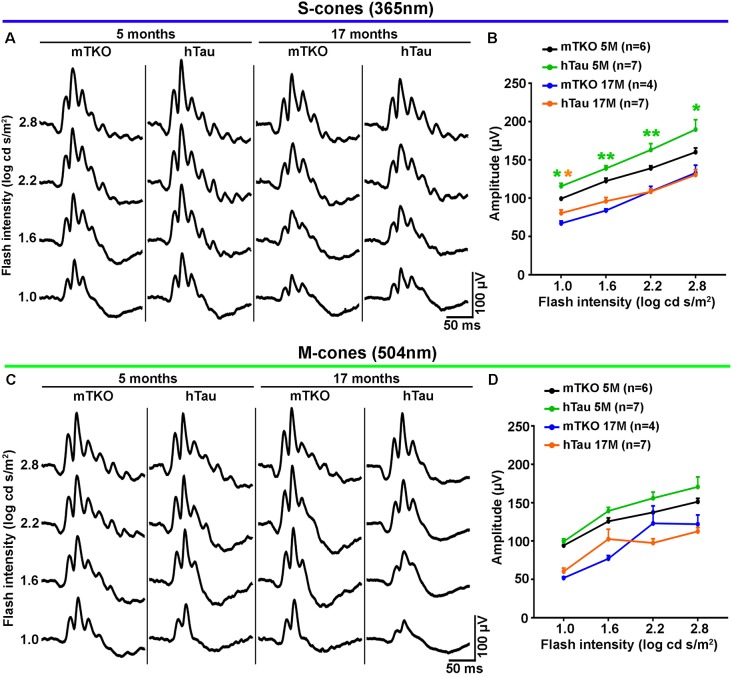
hTau enhances S-cone-mediated photopic electroretinogram (ERG) response. Photopic ERGs were recorded to study the retinal response to light stimulation mediated by cone activation at 5 and 17 months of age. **(A)** Representative ERG responses induced by S-cones activation with increasing intensities of UV light flashes (365 nm). **(B)** Quantitatively, luminance-response function curves of UV light-induced photopic ERGs showed significant differences in b-wave amplitudes between mTKO and hTau mice at 5 months. The b-wave amplitude was higher in hTau than in mTKO mice for light intensities ranging from 1 log cd s/m^2^ to 2.8 log cd s/m^2^. **(C)** Representative ERG responses induced by M-cones stimulation with increasing intensities of green light flashes (504 nm). **(D)** Quantitatively, luminance-response function curves green light-induced photopic ERGs showed no difference between mTKO and hTau b-wave amplitudes at 5 or 17 months. Statistics: *t*-test, unpaired, non-parametric, *P < 0.05, **P < 0.01. Each dot represents mean values ± standard error of the mean (SEM). *n* = 4–7 mice per group.

**Figure 3:**
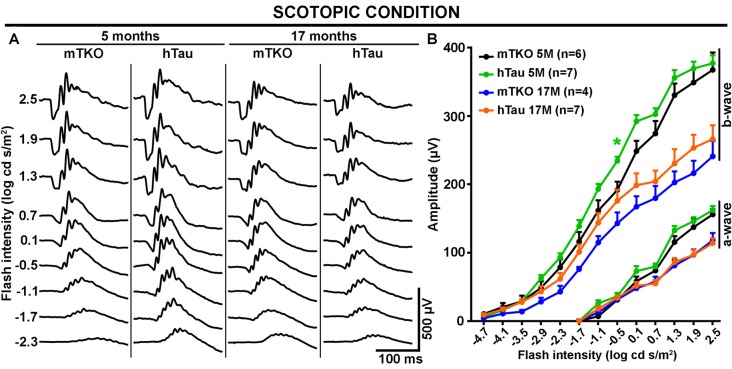
The scotopic ERG response is not altered by hTau expression. The mixed rod-cone ERG response was recorded in scotopic conditions at 5 and 17 months of age in mTKO and hTau mice. **(A)** Representative scotopic ERG waveforms induced by increasing flash intensities. **(B)** Quantitatively, luminance-response function curves revealed similar a- and b-wave amplitudes in the two mouse groups at either age. Nonetheless, the effect of aging was obvious on the a- and b-wave amplitude decline at 17 months when compared with 5 months. Each dot represents mean values ± SEM. *n* = 4–7 mice per group. Statistics: *t*-test, unpaired, *P < 0.05.

**Figure 4:**
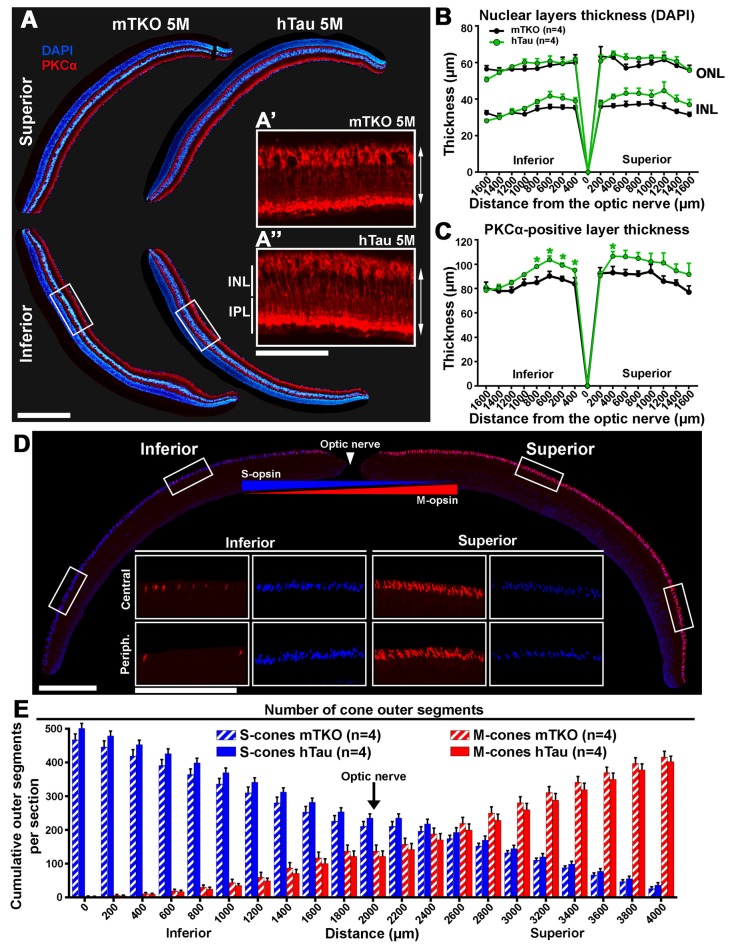
The inner retinal thickness is increased in hTau mice. **(A)** Retinal cross-sections of mTKO and hTau of 5 months of age were stained for PKCα and labeled with DAPI. PKCα-positive bipolar cell bodies were located in the outermost part of the INL and possessed radial extension spanning the INL and the outer plexiform layer. DAPI signal allowed to stain the INL, the ONL and the GCL in the inferior and superior quadrants. **(B)** In 5-month-old mice, the thickness of the INL and ONL were measured every 200 μm from the optic nerve position. The INL and ONL thicknesses were not statistically different in hTau eyes compared with mTKO littermates. **(C)** In 5-month-old mice, the inner retinal thickness was evaluated by measuring the distance between the cell body and the terminal of bipolar cells labeled with PKCα (double arrows in **A’** and **A”**). The bipolar cell layer was significantly thicker in the central retina of hTau than in mTKO mice. **(D)** Immunofluorescent detection of S-opsin and M-opsin in dorso-ventral retinal sections. In cone OS, S-opsin and M-opsin presented opposite dorso-ventral gradients, as previously reported (Haverkamp et al., 2005; Joly et al., 2017). **(E)** Quantitatively, the cumulative number of S- and M-opsin-positive cone OS showed similar gradients in hTau retina to mTKO mice. Statistics: *t*-test, unpaired, non-parametric, *P < 0.05. Each dot and bar represent mean values ± SEM. For quantitative analysis, four sections/mouse were examined in four mice/group. Scale bars: **(A)** = 300 μm and 100 μm, **(D)** = 400 μm and 200 μm. Abbreviations: GCL, ganglion cell layer; INL, inner nuclear layer; IPL, inner plexiform layer; ONL, outer nuclear layer; OS, outer segments.

**Figure 5:**
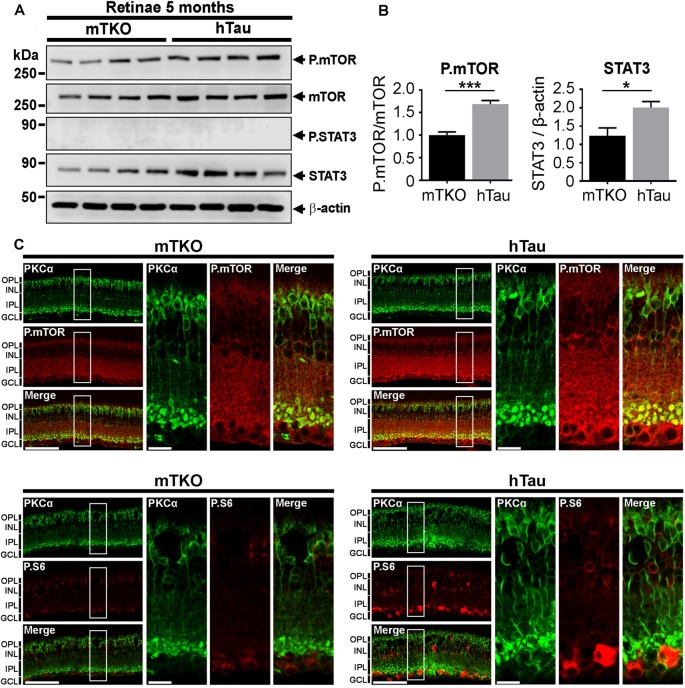
P.mTOR is upregulated in hTau mice. **(A)** Western blot analysis of mTOR and STAT3 activation in retinal lysates. **(B)** Quantitative analysis of Western blots signals with the ImageJ software (NIH) revealed P.mTOR upregulation in hTau mice compared with mTKO animals. **(C)** Increased P.mTOR and P.S6 immunofluorescent signals were detected in the inner retina including bipolar cells stained with PKCα marker in hTau sections. Scale bars: **B** = 200 μm and 100 μm. Statistics: *t*-test, unpaired, ***P < 0.001. *n* = 4 mice/group. Statistics: *t*-test, unpaired, *P < 0.05.

**Figure 6:**
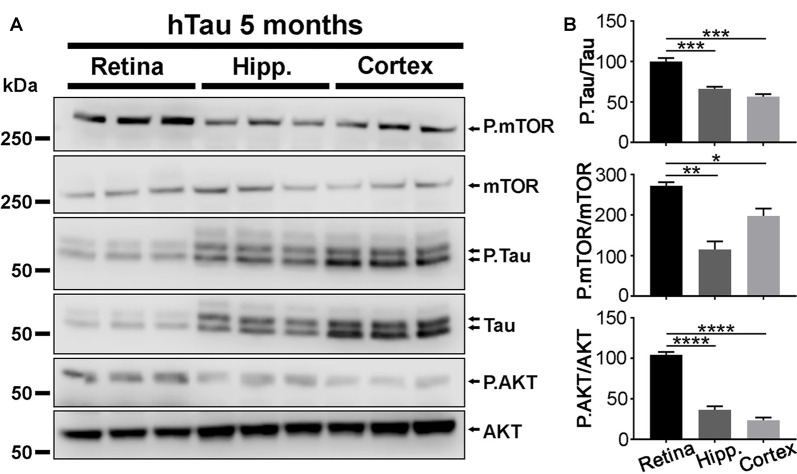
P.Tau expression is higher in the retina than in the brain of hTau mice. **(A)** Western blot analyses were carried out in retinae, hippocampi and cortices of 5 months of age hTau mice. The level of P.Tau, P.mTOR and P.AKT was increased in the retina compared with cerebral structures. **(B)** Quantitative analysis of Western blots signals with the ImageJ software (NIH). The levels of P.Tau, P.mTOR and P.AKT expression were significantly higher in the retina than in brain structures. Statistics: one-way analysis of variance (ANOVA) with Tukey’s *post hoc* test, *P < 0.05, **P < 0.01, ***P < 0.001, ****P < 0.0001. Each bar represents mean values ± SEM. *n* = 3 mice/group.

## Results

### Human Tau Is Expressed in the Inner Retina of hTau Mice

The expression of hTau is sufficient to induce neurological impairments and cell death in the brain of hTau mice (Andorfer et al., 2003; Andorfer, 2005). We speculated that retinal neurons may also express hTau and undergo degeneration in hTau animals. To examine this possibility, the expression level and the distribution of hTau was followed by WB and by IF at 5 months of age. By WB, total Tau and phosphorylated Tau (P.Tau) were detected in hTau retinal lysates using K9JA and PHF1 antibodies, respectively (Figure 1A). The expression of the EGFP reporter allowed to monitor Tau promoter activation. The level of EGFP did not significantly vary between mTKO and hTau mice at 5 months, suggesting similar Tau promoter activity in the two mouse lines. By IF, Tau and P.Tau were highly expressed in inner retinal neurons. The staining of PKCα (Figure 1B) and RBPMS (Figure 1C), that are specific markers for bipolar cells and RGCs respectively, colocalized with Tau and P.Tau. High magnification images allowed to detect Tau in the cytoplasmic compartment of bipolar cells and RGCs. However, a brighter signal for Tau was visualized in RGC axons (Figure 1C) where it is normally bound to microtubules (Tanaka et al., 2015). The distribution of hTau in the retinal cells of hTau mice is similar to that reported for endogenous mouse Tau in WT animals (Chiasseu et al., 2017). Our observations suggest that hTau is expressed in inner retinal cell somata and extensions where it may influence visual information processing.

### hTau Does Not Cause Retinal Dysfunction in Aging Mice but Selectively Enhances S-Cone-Mediated Retinal Activation

As Tau/P.Tau were localized in the cytoplasm of inner nuclear neurons, we wondered if they influenced the function of the hTau retina compared with mTKO mice. To address the functional role of hTau, the retinal response to light stimulation was examined by ERG recordings (Figures 2, 3). In photopic conditions, ERG waveforms are mainly composed of a positive b-wave generated by cells in the inner nuclear layer (INL) and depend on cone photoreceptor activation (Figures 2A–D). Photopic ERGs were obtained in mTKO and hTau mice by selectively activating short-wavelength cones (S-cones) with UV light flashes (365 nm; Figures 2A,B) whereas middle-wavelength cones (M-cones) were activated with green light flashes (504 nm; Figures 2C,D). Strikingly, the ERG b-wave amplitude of hTau eyes was significantly higher than that of mTKO littermates at 5 months of age (Figure 2B; *t*-test, *P < 0.05). This difference was strongly reduced in 17-month-old mice at which age it only persisted for a light intensity of 1.0 log cd s/m^2^ (Figure 2B). With green flashes, only a trend toward an increase was observed in the b-wave amplitude of hTau mice compared with mTKO mice (Figures 2C,D). The retinal function was then analyzed in scotopic conditions. Scotopic ERGs show a negative a-wave resulting from cone and rod photoreceptor activation followed by a b-wave produced by bipolar cells and Müller glia in the inner retinal layer (Figures 3A,B). ERG b-wave measurements only revealed a significant increase in hTau mice relative to mTKO mice at −0.5 log cd s/m^2^ (Figure 3B). The a-wave amplitudes were not different between mTKO and hTau mice (Figure 3B). The luminance-response function curves of 17-month-old mTKO and hTau mice did not differ between each other and were similarly affected by aging when compared to 5-month-old animals (Figure 3B). There was no difference in the peak time of ERG a- and b-waves between mTKO and hTau mice at 5 or 17 months of age (Supplementary Figure S1). Collectively, ERG analysis suggests that hTau does not cause retinal dysfunction in young adult mice and does not exacerbate the effect of aging on retinal function decline. Unexpectedly, hTau significantly enhanced the retinal response mediated by S-cones.

### hTau Influences the Inner Retinal Structure Organization

We set out to assess whether hTau expression affected the retinal structure of mTKO vs. hTau mice. For this purpose, the inner and outer nuclear layers (ONLs) were labeled with the nuclear dye DAPI whereas PKCα was used as a specific marker to stain rod bipolar cells by IF on histological retinal cross-sections (Figure 4A). Inner and ONL thicknesses did not significantly vary between hTau and mTKO mice (Figure 4B). The immunofluorescent signal of PKCα appeared in rod bipolar cell bodies located in the outer margin of the INL and in their radial processes spanning the INL and the inner plexiform layer (IPL; Figure 4A, red). The length of rod bipolar cells was measured to follow variations in the inner retinal thickness (Figure 4B). In the central retina of hTau mice, PKCα-positive cells were longer than in mTKO mice, suggesting a positive structural change induced by hTau in the retina (Figure 4C). The distribution and the number of S-cones and M-cones were followed by IF. As previously reported (Haverkamp et al., 2005; Satoh et al., 2009), the expression pattern of S-opsin and M-opsin showed inversed inferior-superior gradients on mouse retinal sections (Figure 4D). Quantitatively, the escalating number of cones was established from the superior to the inferior retina boundaries. However, the number of labeled S-cones and M-cones did not significantly vary between the two mouse groups at 5-month-old (Figure 4E). By Western blot analysis, the level of constitutively expressed proteins was studied in retinal lysates. Densitometric quantifications of Western blots did not reveal statistical difference in the expression of PKCα, M-opsin (MWL), S-opsin (SWL) and recoverin (rod photoreceptor marker; Supplementary Figure S2). Overall, hTau expression only induces histological changes at the level of rod bipolar cells without affecting other retinal cell types and cell specific markers in hTau mice.

### Phospho-mTOR Is Upregulated in hTau Mouse Retina

Tau has been shown to be a potent modulator of mTOR activation in cell metabolism (Marciniak et al., 2017). We speculated that the enhancement of the inner retina in hTau mice may depend on mTOR upregulation. Indeed, the mTOR signaling pathway is a powerful mechanism controlling retinal cell growth (Park et al., 2008; Cantrup et al., 2012). The influence of hTau on mTOR retinal cell growth was thus monitored by WB in retinal lysates (Figure 5A). The level of P.mTOR was upregulated in hTau compared to mTKO retinae at 5 months of age (Figure 5A). Quantitatively, P.mTOR was significantly increased in hTau retinae (Figure 5B). On retinal cross-sections, the immunofluorescent signal of P.mTOR and P.S6 was stronger between the INL and ganglion cell layer (GCL) of the hTau retina. These observations suggest that hTau enhances P.mTOR activation in hTau retinae in respect with mTKO littermates.

In agreement with previous studies (Andorfer et al., 2003), the thickness of CA1 and that of the DG were smaller in hTau hippocampi than in mTKO controls at 17 months of age, suggesting that hTau induces neurodegeneration in the hippocampus (Supplementary Figure S3). We wondered if the lack of electroretinographic (Figures 2, 3) and histological (Figure 4) alterations in hTau mice was attributable to weaker hTau phosphorylation in the mouse retina than in other CNS tissues such as the hippocampus and the cortex that develop the pathology in hTau mice (Andorfer et al., 2003; Andorfer, 2005; Polydoro et al., 2009). Our Western blot analysis showed that the P.Tau/Tau signal ratio was significantly higher in the retina than in the hippocampus and in the cortex of hTau mice (Figures 6A,B), although the expression level of total Tau was higher in cerebral tissues. The activation of P.mTOR and P.AKT was also stronger in retinal samples than in brain homogenates (Figure 6B). The relatively high level of P.Tau in the retina suggests that the mechanisms controlling Tau phosphorylation are not less active than in affected brain regions, at least, at the level of pSer396/pSer404 (PHF1) and pSer202/pThr205 (AT8).

## Discussion

In the present study, the substitution of mTau by hTau was not toxic for retinal neurons in contrast to what has previously been reported for other CNS neuron populations in the brain (Andorfer et al., 2003). The establishment of a model of transgenic mice expressing hTau was initially aimed at allowing the elucidation of pathological mechanisms underlying AD. We hypothesized that this model may be relevant for the study of AD in the retina as well. Indeed, high level of Tau phosphorylation has been detected in the inner retina of human patients suffering from AD (Doustar et al., 2017). Consistent with this, we observed that hTau was localized in the bipolar cells and in the GCLs of hTau transgenic mice. However, ERG measurements failed to reveal functional alteration at up to 17 months of age. It is important to underline the fact that aging clearly affected the amplitude of ERG a-wave and b-wave between 5 months and 17 months, with or without hTau expression, in a similar fashion to what we (Joly et al., 2017) and others have previously observed (Mazzaro et al., 2016). Other studies revealed that hTau was sufficient to promote neuron degeneration in the mouse brain (Andorfer et al., 2003) and in the eye of the *Drosophila* (Wittmann et al., 2001; Steinhilb et al., 2007). Mouse retinal neurons may thus be relatively resistant to hTau-induced neurodegeneration compared with other neuronal cell populations and with invertebrate neurons. The lack of neurodegeneration in hTau mouse retinae does not seem to be due to the level of Tau phosphorylation that turned out to be higher than in the brain. On the other hand, one cannot exclude that phosphorylation of other Tau residues may differ between the retina and cerebral tissues and thus explain the preservation of the retinal function in hTau animals.

Our data indicate that hTau does not act as a molecular switch triggering AD-associated degeneration in aging retinae. Additional factors may thus be needed to initiate visual deficits in AD, such as Aβ. In this regard, the role played by Aβ in the ocular manifestations of AD is controversial (Ong et al., 2018). For example, we (Joly et al., 2017) and others (Leinonen et al., 2016; Chidlow et al., 2017) did not find retinal dysfunction in APPswe/PS1ΔE9 mice, but the detection of amyloid plaques was reported by Koronyo-Hamaoui et al. (2011). In contrast to the retina, amyloid plaques have consistently been reported in brain structures (Koronyo-Hamaoui et al., 2011; Chidlow et al., 2017; Joly et al., 2017), suggesting a differential susceptibility of retinal vs. cerebral neurons to form amyloid deposits in this mouse model. In the retina, co-expression of hTau and Aβ may be required to induce AD pathogenesis in mice (Ittner et al., 2010). This hypothesis is supported by a recent study showing early pathological changes in 3xTg-AD mouse retina, a transgenic model co-expressing mutant amyloid and Tau genes (Chiasseu et al., 2017). In addition, although hTau did not exert toxic effects in intact mouse retinae in the present study, it may become a potent neurotoxic factor upon lesions. For instance, blast injury enhances the level of P.Tau and its potential toxicity in the retina (Mammadova et al., 2017). RGCs are more prone to develop a tauopathy and to die after mild traumatic injury when they express P301S Tau transgene (Xu et al., 2016). In future studies, it will thus be important to determine if hTau is an injury modulator, increasing the vulnerability of retinal cells to traumatic injuries.

The relatively modest differences of retinal structure and function that were observed between mTKO and hTau mice are in contrast with the strong phenotypic changes reported in the *Drosophila*. In this invertebrate, neuronal development is highly dependent on Tau. For example, progressive degeneration of the eye and the CNS has been observed in flies deprived of Tau (Bolkan and Kretzschmar, 2014). This severe degeneration has been attributed to the fact that Tau is a major microtubule-associated protein (MAP) whose function loss is not compensated by other MAPs (Dehmelt and Halpain, 2005). Moreover, the expression of hTau in the fly induces retinal cell degeneration contrary to what we observed in our study. In fact, Tau expression is dispensable for axonal elongation in developing mice (Harada et al., 1994). The ablation of Tau or the expression of hTau in RGCs did not alter slow and fast axonal transport in the optic nerve (Yuan et al., 2013). The effect of Tau deletion or that of hTau expression may be attenuated by the expression of other MAP such as MAP1B and MAP1A. For instance, MAP1A is upregulated in Tau KO mice and is thought to prevent defects in axon projection development (Harada et al., 1994). Whether compensatory changes in MAP expression occur in mTKO and hTau retinal cell remains to be clarified.

Scotopic ERG measurements suggest that cone-mediated retinal activity is enhanced by hTau expression. A stronger effect of hTau on scotopic ERG response was observed after eye stimulation with UV light than with green flashes. The reason why the response of the blue cone pathway is particularly stronger with hTau is intriguing. Although Tau expression appeared to be weak in mouse photoreceptors in the present study, the phosphorylation of Tau in cones has been proposed to confer resistance to age-associated degeneration in primates (Aboelnour et al., 2017). In some conditions, intracellular expression of hTau may be beneficial for the function and survival of cones. Indeed, for example, hyperphosphorylated hTau has been shown to counteract staurosporine-induced neuronal apoptosis by allowing nuclear translocation of β-catenin (Li et al., 2007). Therefore, the function of Tau is more complex than initially anticipated and is not restricted to AD pathogenesis. Moreover, the ERG gain-of-function that we observed in hTau eyes, relative to mTKO animals, was correlated with longer PKCα-positive bipolar cell extensions. The size of PKCα and non-PKCα-positive bipolar cells may be increased in the INL following a mTOR-dependent mechanism. Indeed, the level of P.mTOR was significantly upregulated in the inner retina of hTau animals relative to mTKO controls. It has recently been reported that interactions between hTau and PTEN resulted in mTOR signaling upregulation (Marciniak et al., 2017). In a similar manner, hTau may relieve PTEN inhibition on mTOR and hence activate protein and lipid synthesis and increase the thickness of retinal cell layers. This hypothesis is supported by the results of Cantrup et al. (2012) showing that PTEN deletion leads to mTOR signaling upregulation and retinal cell hypertrophy in mice. However, in contrast to the functional improvement that we observed, uncontrolled mTOR signaling activation altered retinal cytoarchitecture and function in the study of Cantrup and co-workers. In this case, the detrimental effects caused by PTEN gene deletion may be attributable to mTOR over-activation during retinal development, presumably at a stimulation level that is not reached in hTau-expressing mice. Consequently, our data suggest that Tau is a physiological regulator of mTOR signaling that may be useful for improving vision in retinal diseases such as ischemic retinopathies. This possibility deserves to be further tested in pathological conditions.

In conclusion, this study demonstrates that hTau expression is not detrimental for retinal neurons in contrast to what has been reported for brain neurons. Interestingly, our results also reveal that hTau can exert positive effects on the function and structure of the retina, possibly by upregulating mTOR signaling. The modulation of the hTau/mTOR signaling in retinal cell survival and repair may allow the development of new therapeutic strategies for ocular diseases.

## Author Contributions

VP designed the experimental plan of the study. LR performed all experiments and data analysis contained in the study under the supervision of VP with the technical help of SJ and with a contribution of JM. MB-L performed part of the IF staining. LR and VP wrote the manuscript. LR, SJ, JM, EP and VP revised the manuscript critically. All authors have given their approval to the final version of the manuscript.

## Conflict of Interest Statement

The authors declare that the research was conducted in the absence of any commercial or financial relationships that could be construed as a potential conflict of interest.
