# Corrigendum: Human Tau Expression Does Not Induce Mouse Retina Neurodegeneration, Suggesting Differential Toxicity of Tau in Brain vs. Retinal Neurons

**DOI:** 10.3389/fnmol.2018.00390

**Published:** 2018-10-24

**Authors:** Léa Rodriguez, Julius Baya Mdzomba, Sandrine Joly, Mélissa Boudreau-Laprise, Emmanuel Planel, Vincent Pernet

**Affiliations:** ^1^CUO-Recherche, Centre de Recherche du CHU de Québec, Quebec, QC, Canada; ^2^Département d'ophtalmologie, Faculté de Médecine, Université Laval, Quebec, QC, Canada; ^3^Axe Neurosciences, Centre de Recherche du CHU de Québec, Quebec, QC, Canada; ^4^Département de Psychiatrie et de Neurosciences, Faculté de Médecine, Université Laval, Quebec, QC, Canada

**Keywords:** human Tau, Alzheimer's disease, retina, electroretinogram, photoreceptors, bipolar cells, mTOR

In the original article, the reference for (Leinonen et al., 2016) was incorrectly written as Leinonen, H., Keksa-Goldsteine, V., Ragauskas, S., Kohlmann, P., Singh, Y., Savchenko, E., et al. (2017). Retinal Degeneration in a mouse model of CLN5 disease is associated with compromised autophagy. *Sci. Rep*. 7:1597. doi: 10.1038/s41598-017-01716-1.

It should be Leinonen, H., Lipponen, A., Gurevicius, K., and Tanila, H. (2016). Normal amplitude of electroretinography and visual evoked potential responses in AβPP/PS1 mice. *J. Alzheimers Dis*. 51, 21–26. doi: 10.3233/JAD-150798.

The authors apologize for this error and state that this does not change the scientific conclusions of the article in any way. The original article has been updated.

## Conflict of interest statement

The authors declare that the research was conducted in the absence of any commercial or financial relationships that could be construed as a potential conflict of interest.
