# Supplementary material for: Human Tau Expression Does Not Induce Mouse Retina Neurodegeneration, Suggesting Differential Toxicity of Tau in Brain vs. Retinal Neurons
Source: Front Mol Neurosci. 2018 Aug 24;11:293. doi: 10.3389/fnmol.2018.00293 (PMC6117378; doi:10.3389/fnmol.2018.00293)
Supplement: Supplementary file 1 [file Data_Sheet_1.docx]

**Supplementary materials**

FIGURE S1


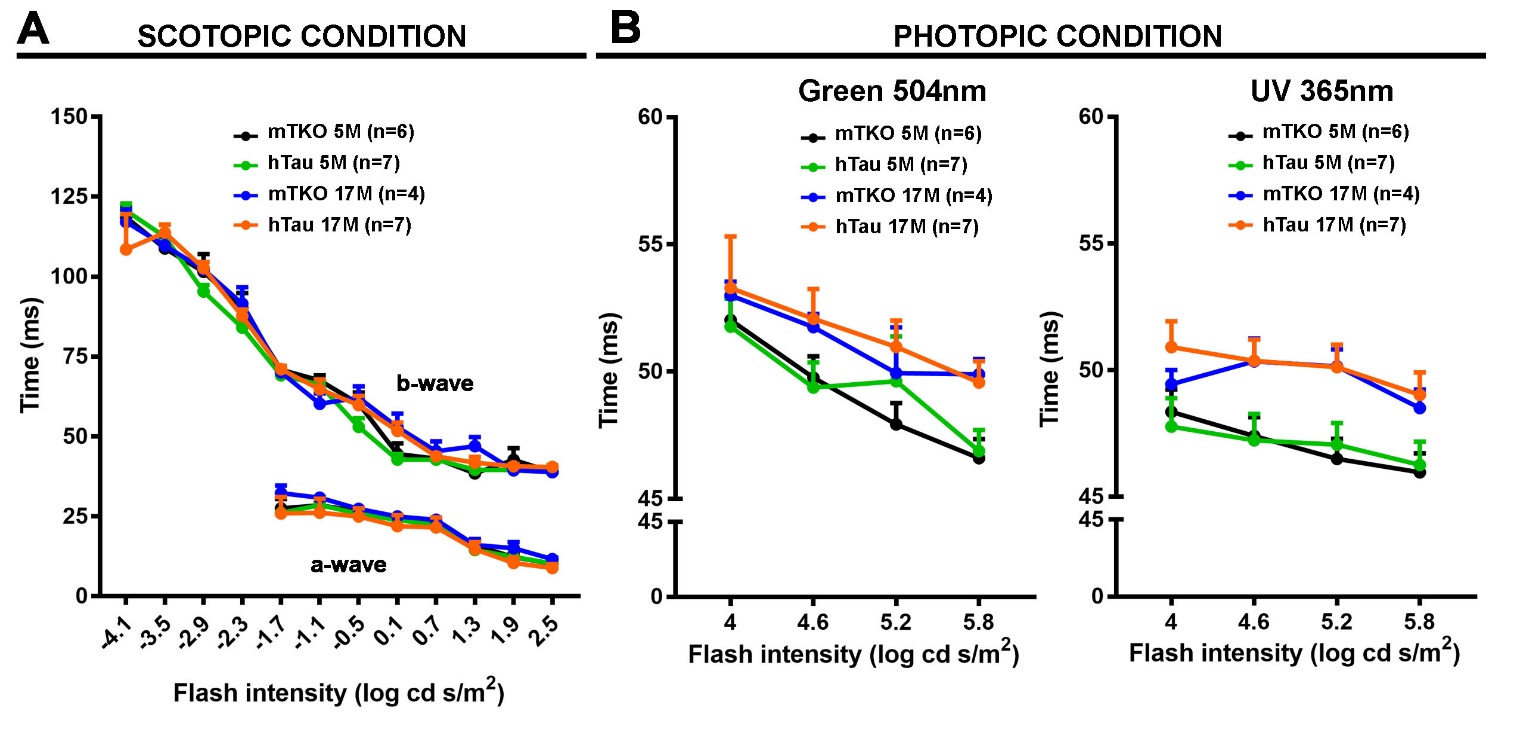


**Figure S1. hTau does not influence ERG peak times in scotopic and photopic conditions.**

Peak times of ERG a-waves and b-waves presented in Figures 2 and 3 were analyzed.

**A.** Quantitatively, luminance-peak time function curves showed no significant difference between mTKO and hTau b-wave latencies at 5 or 17 months in scotopic condition.

**B.** Quantitatively, luminance-peak time function curves showed no difference between mTKO and hTau b-wave latencies at 5 or 17 months in photopic conditions (green and UV).

Each dot represents mean values ± SEM. n = 4-7 mice per group.

FIGURE S2


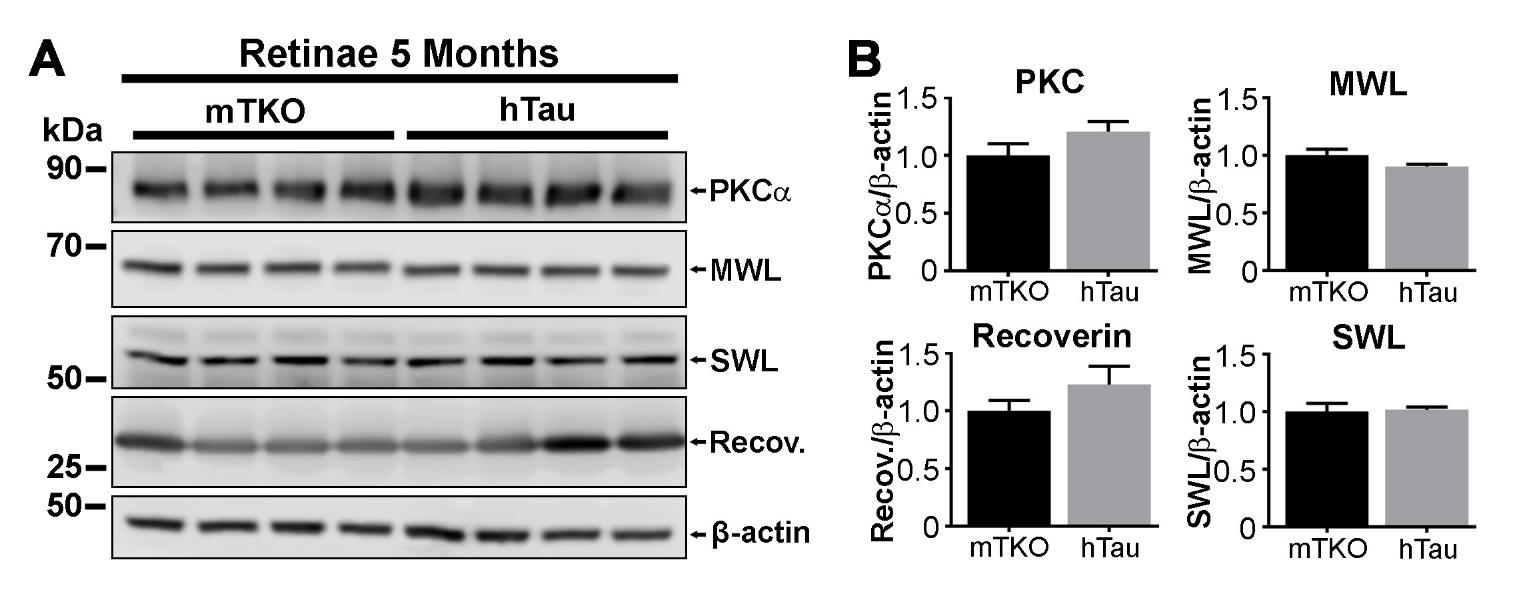


**Figure S2. hTau does not influence the level of constitutively expressed retinal proteins.**

**A.** Western blot analysis of cell-specific markers in 5-month-old mTKO and hTau retinae, PKCα (bipolar cells), MWL (M-cones), SWL (S-cones), Recoverin (rods).

**B.** Quantitative measurements of Western blot signals with the image J software (NIH). The level of constitutively expressed proteins did not vary between the 2 experimental groups.

Each bar represents mean values ± SEM. n = 4 mice per group.

FIGURE S3


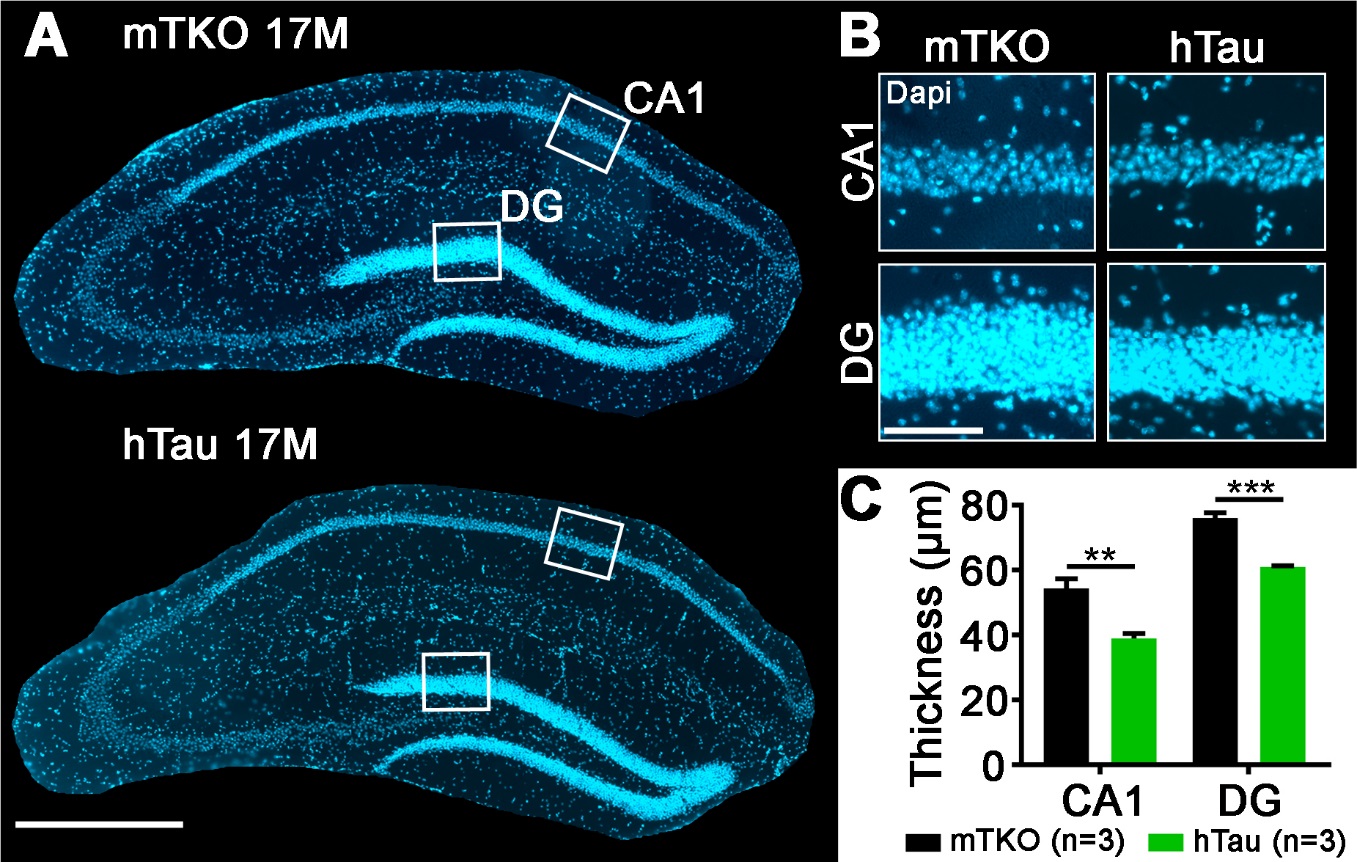


**Figure S3. hTau expression induces hippocampal neurodegeneration in 17-month-old mice.**

**A.** Representative images of 17-month-old mTKO vs hTau mouse brain sections stained with Dapi.

**B.** Close-up of CA1 and DG cell layers of mTKO and hTau mice.

**C.** Thickness analysis of CA1 and DG in 17-month-old mTKO vs hTau mouse hippocampal sections.

Data represent mean values ± S.E.M. n = 3 mice per group.

Statistics: non-parametric unpaired t-test, **: P < 0.01; ***: P < 0.001.

Scale bars: A = 500 µm and B = 100 µm.
